# Ginsenoside-Rh2 inhibits U251 glioma cell migration and invasion via the Gab2/Akt2 pathway

**DOI:** 10.3389/fonc.2026.1744705

**Published:** 2026-04-01

**Authors:** Wei Sun, Ruifang Li, Linjuan Wang, Wenjie Han, Jiake Li, Shuangli Xu, Xiuning Sun

**Affiliations:** 1 Department of Emergency Medicine, Affiliated Hospital of Shandong Second Medical University, Weifang, China; 2 Department of Pathogenic Biology, Shandong Second Medical University, Weifang, China; 3 Department of Rehabilitation, Weifang Hospital of Traditional Chinese Medicine, Weifang, China; 4 Hemodialysis Room, Affiliated Hospital of Shandong Second Medical University, Weifang, China; 5 Qingdao Medical College, Qingdao University, Qingdao, China

**Keywords:** Akt2, Gab2, ginsenoside-Rh2, glioma, invasion

## Abstract

**Background:**

Glioma, the most common primary intracranial tumor, exhibits high recurrence and mortality rates. Ginsenoside-Rh2 (GS-Rh2), an active compound from *Panax ginseng*, has shown anti-tumor potential. Gab2, a tyrosine kinase substrate, is implicated in glioma pathogenesis; however, the mechanism by which GS-Rh2 might inhibit glioma cell migration and invasion via the Gab2/Akt2 pathway remains unexplored.

**Objective:**

To investigate the effects and mechanisms of GS-Rh2 on glioma cell migration and invasion through the Gab2/Akt2 signaling pathway.

**Methods:**

U251 glioma cells were treated with GS-Rh2 *in vitro*. Cell viability was assessed by MTT assay. Western blot was used to detect Gab2 and p-Akt2 expression. *In vivo*, GS-Rh2 was administered to nude mice bearing U251 or siGab2/U251 intracranial xenografts. HE staining assessed brain invasion, and western blot detected Gab2 and Akt2 expression in tumor tissues.

**Results:**

GS-Rh2 significantly inhibited U251 cell proliferation, migration, and invasion both *in vitro* and *in vivo* (*P* < 0.05), while no significant effects were observed in siGab2/U251 cells. At the molecular level, GS-Rh2 significantly reduced Gab2 expression and Akt2 phosphorylation in U251 cells and brain tumor tissues (*P* < 0.05).

**Conclusions:**

GS-Rh2 inhibits migration and invasion of U251 glioma cells by decreasing Gab2 expression and Akt2 phosphorylation, suggesting that GS-Rh2 targets the Gab2/Akt2 signaling axis.

## 1 Introduction

Gliomas are the most prevalent primary intracranial neurological tumors, characterized by high rates of recurrence and mortality (1–3). Their progressive and aggressive growth pattern, coupled with the absence of a distinct boundary between tumor and normal tissue, renders complete surgical resection exceedingly difficult (1–3). This has prompted increasing research into the molecular mechanisms underlying glioma invasion.

Ginsenoside-Rh2 (GS-Rh2), a naturally occurring active compound extracted from ginseng, exhibits low toxicity and readily crosses the blood-brain barrier (4). Numerous studies have demonstrated the significant potential of GS-Rh2 in preventing and treating various cancers (4–7). GS-Rh2 has been shown to inhibit proliferation of multiple tumor cell lines (4, 6) and to prevent metastasis and invasion in colon, thyroid, and breast cancers (8–10). However, the mechanisms by which GS-Rh2 inhibits glioma migration and invasion remain relatively unexplored.

Grb-associated binding protein 2 (Gab2) is a scaffold/docking protein that binds to tyrosine kinases, mediating the coupling of membrane receptors to signal transducers. This process enables mutual regulation of various signaling proteins and rapid signal amplification (11–13). Recent studies have indicated that Gab2 possesses proto-oncogenic properties, and its expression is strongly correlated with invasion and metastasis in a range of tumors, including gliomas (14–21).

Akt2, a prominent serine/threonine kinase isoform, plays a crucial role in regulating cell proliferation and differentiation (22, 23). Moreover, Akt2 expression is strongly associated with tumor cell proliferation and metastasis (24, 25), including a significant link to metastatic invasion in gliomas (26, 27). Studies employing small RNA interference technology to suppress Akt2 expression in malignant glioma cells have shown that silencing the Akt2 gene reduces the motility and invasive capacity of these cells by decreasing F-actin polymerization and impairing adhesion to the extracellular matrix (28).

Previous studies (29) have demonstrated that Gab2 is highly expressed in glioma tissues and that reducing Gab2 expression inhibits Akt2 phosphorylation, consequently suppressing glioma cell migration and invasion. This is consistent with findings demonstrating a strong correlation between glioblastoma invasion and the expression of both Gab2 and Akt2 (30). These results suggest that Gab2 may serve as a novel therapeutic target for inhibiting glioma invasion and metastasis.

While prior work has independently established the anti-tumor properties of GS-Rh2 and the involvement of the Gab2/Akt2 axis in glioma biology, no study has investigated whether GS-Rh2 can modulate glioma invasion specifically through the Gab2/Akt2 pathway. The novelty of the present study lies in directly linking GS-Rh2 treatment to the suppression of the Gab2/Akt2 signaling axis, providing mechanistic evidence that GS-Rh2 exerts its anti-invasive effects on glioma cells through this specific pathway. This study aims to elucidate this mechanism through both *in vivo* and *in vitro* experiments, providing experimental and theoretical support for the clinical application of GS-Rh2 in glioma therapy.

## 2 Materials and methods

This experiment was approved by the Animal Ethics Committee of Shandong Second Medical University (No. 2025SDL784).

### 2.1 *In vitro* experiments

#### 2.1.1 Cell culture and grouping

To investigate the role of Gab2 in U251 cells (ATCC, USA), RNA interference (RNAi) technology was employed to downregulate Gab2 expression. The Gab2-targeting shRNA sequence (5′-GCAGCACAGTTCCTTCATA-3′) was designed using siDirect v2.0 software and synthesized by GenePharma Co., Ltd. (Shanghai, China). The shRNA was cloned into the pGPU6/GFP/Neo vector and transfected into U251 cells using Lipofectamine 3000 (Invitrogen, USA) according to the manufacturer’s protocol. Stable clones were selected with G418 (400 µg/mL, Sigma-Aldrich, USA) for 4 weeks. A non-targeting scrambled shRNA (5′-TTCTCCGAACGTGTCACGT-3′) served as a negative control. Knockdown efficiency was confirmed by both quantitative real-time PCR (qRT-PCR) and western blot analysis, which demonstrated approximately 75% reduction of Gab2 mRNA and 70% reduction of Gab2 protein expression in siGab2/U251 cells compared to parental U251 cells (*P* < 0.01), consistent with our previously published data (29).

The cells were cultured in RPMI 1640 medium (HyClone, USA) supplemented with 10% fetal bovine serum (FBS, HyClone, USA) in a 5% CO_2_ incubator at 37 °C. The experimental groups consisted of U251, U251 + GS-Rh2 (GS-Rh2, C_36_H_62_O_8_, molecular weight 623; Zhejiang Yak Pharmaceutical Co., Ltd.), siGab2/U251, and siGab2/U251 + GS-Rh2.

#### 2.1.2 Determination of IC50 of GS-Rh2 by MTT assay

GS-Rh2 was prepared as a stock solution at 10 µg/mL using a DMSO/ethanol mixture (1:5, v/v). The final DMSO concentration in the culture medium was less than 0.1% (v/v), which has no detectable cytotoxic effect. Cells in the logarithmic growth phase were seeded into 96-well culture plates at a density of 5 × 10^3^ cells/well in a volume of 200 µL per well. Different drug concentration groups were established at 5, 10, 20, 40, and 80 µg/mL. A vehicle control group received only the drug diluent (DMSO/ethanol at equivalent volume). Each concentration group had 5 replicate wells.

After incubation at 37 °C in a 5% CO_2_ incubator for 24 hours, 10 µL of MTT working solution (5 mg/mL) was added to each well. The cells were further incubated for 4 hours, followed by careful removal of the culture supernatant. Then, 200 µL of DMSO was added to each well, and the absorbance at 492 nm was measured using a microplate reader.

#### 2.1.3 Assessment of cell proliferation by MTT assay

Cells were seeded into 96-well culture plates at a density of 5 × 10^3^ cells/well, with 5 replicate wells per group. After 24 hours of adhesion culture, the U251 + GS-Rh2 and siGab2/U251 + GS-Rh2 groups were treated with GS-Rh2 at the IC50 concentration, while the other two groups received equivalent vehicle (DMSO/ethanol). After 24 hours of drug treatment, MTT assay was performed as described above. Growth inhibition rate was calculated as: Inhibition rate = (OD_control_ – OD_experimental_)/OD_control_ × 100%.

#### 2.1.4 Western blot analysis of Gab2 and Akt2 expression

Cells were seeded into 6-well plates at a density of 2 × 10^5^ cells/mL, with the same grouping and drug treatment as described above. After 24 hours, cells were lysed with RIPA buffer containing PMSF (1 mM). Total protein was extracted after incubation on ice for 30 minutes, and protein concentration was determined by BCA assay (Beyotime, China). Equal amounts of protein (30 µg per lane) were separated by 10% SDS-PAGE and transferred onto PVDF membranes (Millipore, USA).

The membranes were blocked with 5% skim milk in TBST for 1 hour at room temperature. Primary antibodies against Gab2 (1:1000, rabbit polyclonal, Santa Cruz Biotechnology, USA), Akt2 (1:1000, rabbit polyclonal, Santa Cruz Biotechnology, USA), p-Akt2 (Ser474; 1:1000, rabbit polyclonal, Santa Cruz Biotechnology, USA), and β-actin (1:2000, mouse monoclonal, Santa Cruz Biotechnology, USA) were diluted in TBST containing 5% skim milk and incubated with the membranes overnight at 4 °C. After washing, membranes were incubated with HRP-conjugated secondary antibody (goat anti-rabbit IgG, 1:5000, Abcam, USA) at room temperature for 60 minutes. Protein bands were visualized using an ECL detection system and captured with a ChemiDoc imaging system (Bio-Rad, USA). Band intensities were quantified by densitometry using ImageJ software (National Institutes of Health, USA), and relative expression was normalized to β-actin (for Gab2) or total Akt2 (for p-Akt2). Each experiment was performed independently at least three times (n = 5 per group).

#### 2.1.5 Assessment of tumor cell migration

Cells were pretreated with GS-Rh2 for 0.5 hours and then suspended in serum-free RPMI 1640 medium (HyClone, USA) at a density of 5 × 10^5^/mL. A 100 µL aliquot of cell suspension was added to the upper chamber of an 8.0 µm Transwell insert (Neuroprobe, USA). The lower chamber contained 500 µL of medium supplemented with 2.5% FBS. Cells were cultured at 37 °C in 5% CO_2_ for 12 hours. Cells remaining on the upper surface of the membrane were removed with a wet cotton swab. Cells that migrated to the lower surface were fixed with 4% formaldehyde, washed with PBS, stained with DAPI, and counted under a fluorescence microscope. The number of migrated cells reflects the migration ability of the tumor cells. Each experiment was repeated three times, and the results were averaged.

#### 2.1.6 Assessment of tumor cell invasion

An 8.0 µm Transwell insert was coated with 1.5 mg/mL Matrigel (BD Biosciences, USA) on the upper compartment membrane. After drug pretreatment for 0.5 hours, cells were suspended in serum-free RPMI 1640 medium at a density of 5 × 10^5^/mL. The cell suspension was added to the upper chamber, and medium containing 5% FBS was added to the lower chamber. After 12 hours of culture, cells and Matrigel on the upper surface were removed. The membrane was washed with PBS, and cells on the lower surface were fixed with 100% methanol and stained with DAPI for 5–10 minutes. Cell counts were performed under a fluorescence microscope. Each experiment was repeated three times, and the results were averaged.

### 2.2 *In vivo* experiments

#### 2.2.1 Experimental grouping for intracranial glioma model

Six-week-old male BALB/c nude mice (nu/nu, 18–22 g) were purchased from Beijing Vital River Laboratory Animal Technology Co., Ltd. All animal procedures followed the Guide for the Care and Use of Laboratory Animals of the National Institutes of Health and abided by the ARRIVE guidelines. The mice were randomly assigned to four groups (n = 12 per group):

Group I (U251 control): intracranial inoculation of U251 cell suspension (1 × 10^6^ cells/mL, 10 µL) with intraperitoneal injection of 0.1% DMSO vehicle every other day for 6 weeks. Group II (U251 + GS-Rh2): intracranial inoculation of U251 cells as above with intraperitoneal injection of GS-Rh2 (10 mg/kg) every other day for 6 weeks. This dosage was selected based on previous pharmacological studies demonstrating effective anti-tumor activity of GS-Rh2 at 5–20 mg/kg in murine models (7, 31), and pharmacokinetic evidence indicating that intraperitoneally administered GS-Rh2 achieves detectable concentrations in brain tissue owing to its ability to cross the blood-brain barrier (4, 32). Group III (siGab2/U251): intracranial inoculation of siGab2/U251 cells (1 × 10^6^ cells/mL, 10 µL) with vehicle injection as in Group I. Group IV (siGab2/U251 + GS-Rh2): intracranial inoculation of siGab2/U251 cells with GS-Rh2 treatment as in Group II.

#### 2.2.2 Establishment of intracranial glioma model

Nude mice were anesthetized by intraperitoneal injection of 0.3% sodium pentobarbital (50 mg/kg) and fixed in a small-animal stereotactic frame (RWD Life Science, China). A midline scalp incision was made, and a burr hole was drilled at 2 mm lateral to the right sagittal suture and 1 mm anterior to the bregma. Cell suspension (10 µL) was injected at a depth of 3 mm below the dural surface into the right caudate nucleus using a 10-µL Hamilton microsyringe at a rate of 1 µL/min. After injection, the needle was left in place for 5 minutes before slow withdrawal to minimize reflux. The scalp was sutured, and mice were monitored daily for neurological signs.

#### 2.2.3 HE staining

Six weeks after cell inoculation, mice were euthanized by intraperitoneal injection of sodium pentobarbital overdose. A portion of the brain tissue was fixed in 4% paraformaldehyde for 24 hours, followed by dehydration and paraffin embedding. Coronal sections (5 µm) were stained with hematoxylin and eosin (HE). The number of satellite tumor nodules surrounding the main tumor mass was counted under a light microscope. For each group, 24 sections were randomly selected, and the average number of satellite nodules per section was calculated.

#### 2.2.4 Western blot analysis of brain tumor tissue

Six weeks after cell inoculation, mice were euthanized and brain tumor tissue was collected for total protein extraction. Western blot analysis was performed as described in Section 2.1.4 to determine the expression levels of Gab2 and the phosphorylation status of Akt2.

#### 2.2.5 Subcutaneous tumor assessment

To further evaluate the growth-inhibitory effect of GS-Rh2, subcutaneous xenografts were established by injecting U251 or siGab2/U251 cells into the right flank of nude mice. After 6 weeks of treatment, mice were euthanized, tumors were carefully excised, and tumor weight was recorded.

### 2.3 Statistical analysis

All data were analyzed using SPSS 17.0 (IBM Corp., USA) and are expressed as mean ± standard deviation. Data were first assessed for normal distribution using the Shapiro–Wilk test and for homogeneity of variance using Levene’s test. As all datasets met the assumptions of normality and equal variance, one-way analysis of variance (ANOVA) was performed for comparisons among multiple groups, followed by the least significant difference (LSD) test for pairwise comparisons. A significance level of *P* < 0.05 was considered statistically significant. All experiments were performed independently at least three times unless otherwise specified.

## 3 Results

### 3.1 Determination of IC50 of GS-Rh2

The MTT assay revealed a dose-dependent inhibition of U251 cell viability by GS-Rh2 at concentrations of 5, 10, 20, 40, and 80 µg/mL. Using SPSS 17.0, the IC50 of GS-Rh2 on U251 cells was calculated to be 31 µg/mL. This concentration was subsequently used for all experiments.

### 3.2 GS-Rh2 inhibited proliferation of U251 cells, reduced Gab2 expression, and suppressed Akt2 phosphorylation

**Figure 1:**
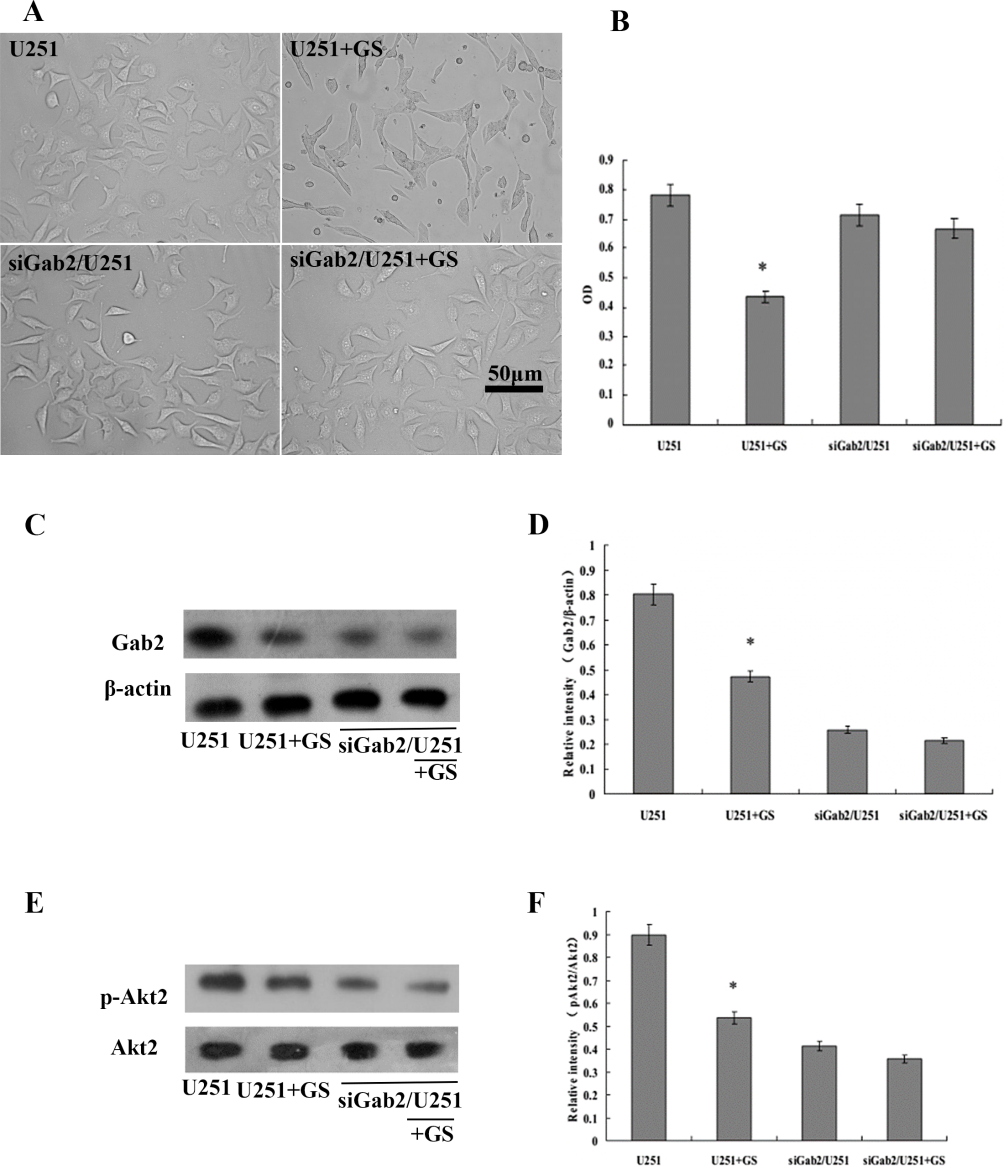
GS-Rh2 inhibits U251 cell proliferation, reduces Gab2 expression, and suppresses Akt2 phosphorylation *in vitro*. **(A)** Representative cell morphology images of each group (×200). Cells in the U251, siGab2/U251, and siGab2/U251 + GS-Rh2 groups exhibited robust adherent growth with uniform size, clear boundaries, star-shaped branching, and good refractive properties. In contrast, cells in the U251 + GS-Rh2 group appeared shrunken and rounded, with increased intracytoplasmic particles, reduced attachment, and disordered morphology. **(B)** Cell viability (OD values) measured by MTT assay. GS-Rh2 significantly reduced U251 cell viability (n = 5, *P < 0.05 vs. U251 group, LSD test), while no significant effect was observed in siGab2/U251 cells. **(C)** Representative western blot images of Gab2 protein expression, with β-actin as the loading control. **(D)** Densitometric quantification of Gab2 protein expression relative to β-actin (n = 5, *P < 0.05 vs. U251 group, LSD test). **(E)** Representative western blot images of p-Akt2 and total Akt2 protein expression. **(F)** Densitometric quantification of p-Akt2 relative to total Akt2 (n = 5, *P < 0.05 vs. U251 group, LSD test).

After 24 hours of treatment with GS-Rh2 at the IC50 concentration (31 µg/mL), the proliferation of U251 cells was significantly inhibited compared to the control group (n = 5, *P* < 0.05, LSD *post-hoc* test). In contrast, there was no significant difference in OD values between the siGab2/U251 group and the siGab2/U251 + GS-Rh2 group (*P* > 0.05), indicating that GS-Rh2 did not significantly affect the proliferation of siGab2/U251 cells (**Figures 1A, B**).

Western blot analysis and densitometric quantification using ImageJ revealed that Gab2 expression in the U251 + GS-Rh2 group was significantly reduced compared to the U251 group (n = 5, *P* < 0.05). Gab2 expression in both the siGab2/U251 and siGab2/U251 + GS-Rh2 groups was low, with no significant difference between them (*P* > 0.05), indicating that GS-Rh2 had no additional inhibitory effect on Gab2 expression in cells already depleted of Gab2 (**Figures 1C, D**).

Furthermore, the phosphorylation level of Akt2 (p-Akt2/total Akt2) in the U251 + GS-Rh2 group was significantly decreased compared to the control group (n = 5, *P* < 0.05). The p-Akt2 levels in the siGab2/U251 and siGab2/U251 + GS-Rh2 groups were similarly low, with no significant difference between them (*P* > 0.05). Total Akt2 protein levels remained relatively stable across all four groups, confirming that the observed changes were specific to Akt2 phosphorylation rather than total Akt2 expression (**Figures 1E, F**).

### 3.3 GS-Rh2 inhibited migration and invasion of U251 cells

**Figure 2:**
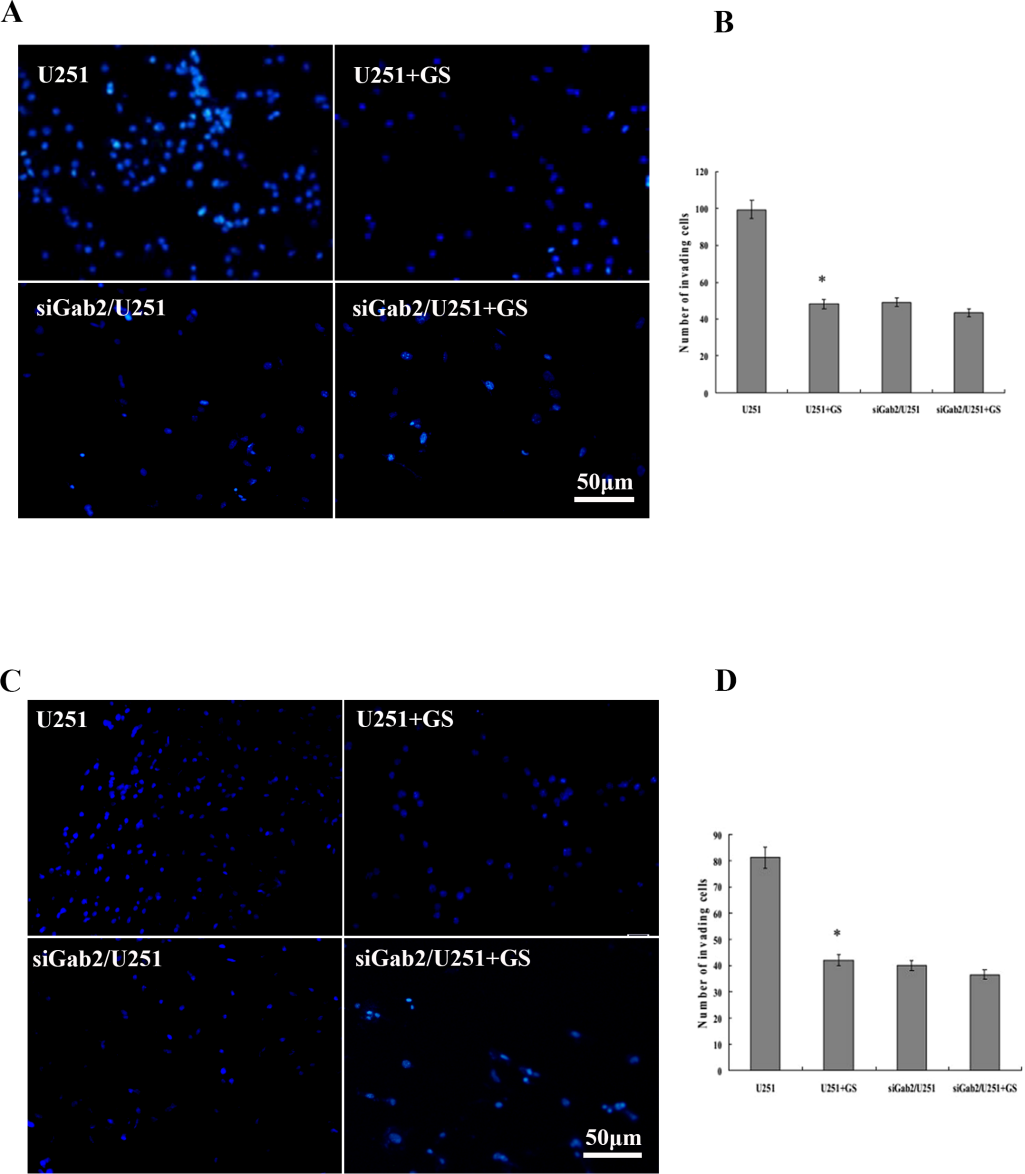
GS-Rh2 inhibits migration and invasion of U251 cells *in vitro*. **(A)** Representative images of migrated cells on the lower surface of Transwell membranes (×200, DAPI staining). **(B)** Quantification of migrated cells per field (n = 5, *P < 0.05 vs. U251 group, LSD test). **(C)** Representative images of invaded cells that penetrated the Matrigel-coated Transwell membrane (×200, DAPI staining). **(D)** Quantification of invaded cells per field (n = 5, *P < 0.05 vs. U251 group, LSD test).

In the Transwell migration assay, the U251 group exhibited strong migration ability. GS-Rh2 treatment significantly reduced the number of migrated cells in the U251 + GS-Rh2 group compared to the U251 group (n = 5, *P* < 0.05). The siGab2/U251 and siGab2/U251 + GS-Rh2 groups both showed lower cell migration, with no significant difference between them (*P* > 0.05). Notably, the migration ability of the siGab2/U251 group was significantly lower than that of the U251 group, confirming that Gab2 knockdown itself reduces cell migration, consistent with our previous findings (29) (**Figures 2A, B**).

In the Matrigel invasion assay, the number of U251 cells penetrating the extracellular matrix was significantly reduced after GS-Rh2 treatment compared to the control (n = 5, *P* < 0.05). The siGab2/U251 and siGab2/U251 + GS-Rh2 groups showed similarly low invasion ability, with no significant difference between them (*P* > 0.05). These results indicate that GS-Rh2 did not exert additional inhibitory effects on invasion after Gab2 was already knocked down, consistent with our previous findings (29) (**Figures 2C, D**).

### 3.4 GS-Rh2 inhibited glioma invasion in the brain of nude mice

**Figure 3:**
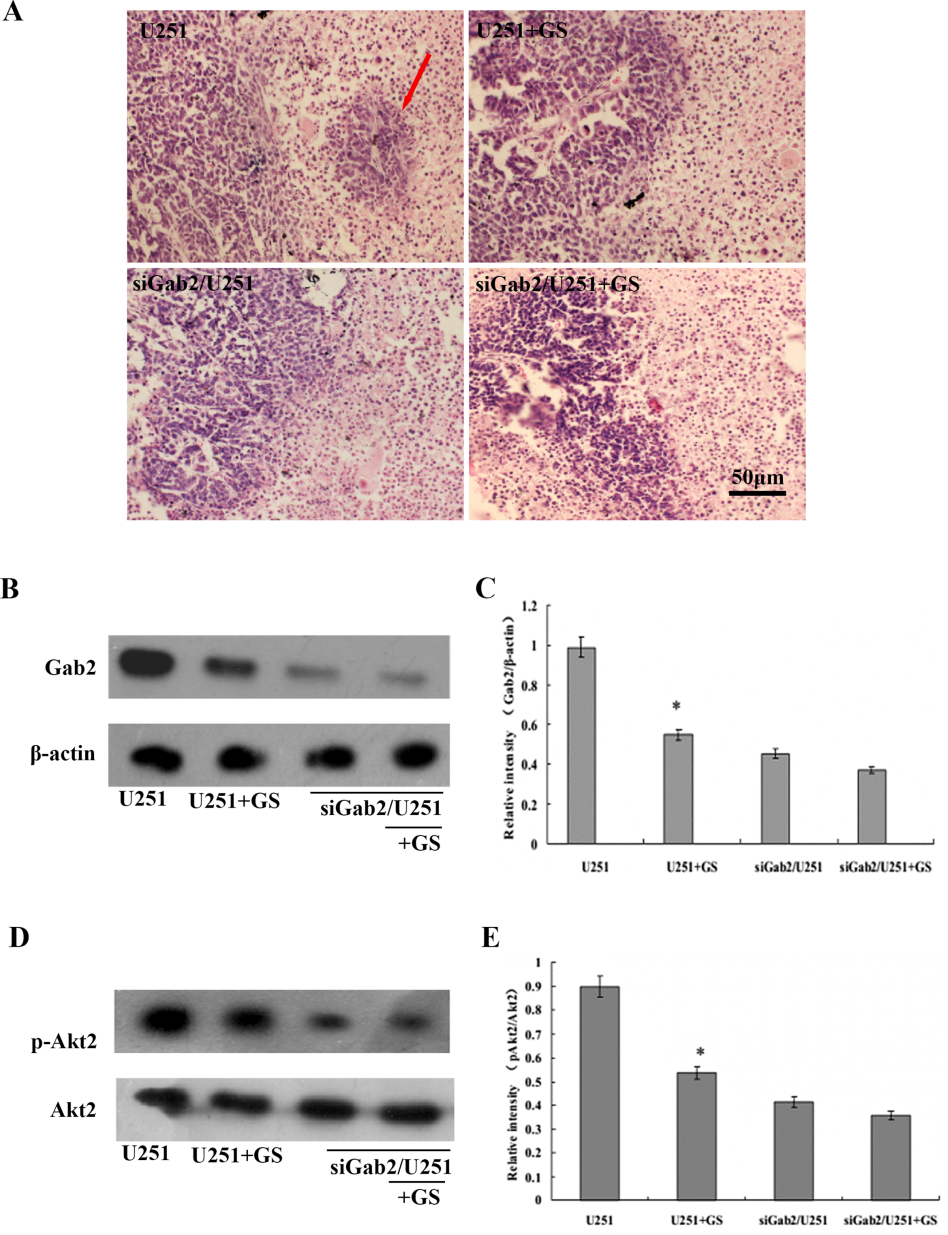
GS-Rh2 inhibits glioma invasion, Gab2 expression, and Akt2 phosphorylation in the brain of nude mice. **(A)** Representative HE-stained coronal brain sections showing satellite tumor nodules surrounding the main tumor mass in each group. Red arrow indicates satellite nodules in the U251 group; black arrow indicates reduced satellite nodules in the U251 + GS-Rh2 group. **(B)** Representative western blot images of Gab2 protein expression in brain tumor tissues, with β-actin as the loading control. **(C)** Densitometric quantification of Gab2 expression relative to β-actin (n = 5, *P < 0.05 vs. U251 group, LSD test). **(D)** Representative western blot images of p-Akt2 and total Akt2 in brain tumor tissues. **(E)** Densitometric quantification of p-Akt2 relative to total Akt2 (n = 5, *P < 0.05 vs. U251 group, LSD test).

HE staining of brain tissue sections revealed that the number of satellite tumor nodules around the main tumor mass was markedly reduced in the U251 + GS-Rh2 group compared to the U251 control group (n = 5, *P* < 0.05), suggesting that GS-Rh2 effectively inhibited glioma invasion in the brain. The siGab2/U251 group also showed fewer satellite nodules than the U251 group, consistent with our previous results (29). GS-Rh2 did not significantly further reduce the invasion of siGab2/U251 cells (**Figure 3A**).

### 3.5 GS-Rh2 inhibited Gab2 expression and Akt2 phosphorylation in intracranial gliomas

Western blot analysis of brain tumor tissues revealed that compared to the U251 group, Gab2 protein expression and Akt2 phosphorylation levels were significantly decreased in the U251 + GS-Rh2, siGab2/U251, and siGab2/U251 + GS-Rh2 groups (n = 5, *P* < 0.05, LSD *post-hoc* test) (**Figures 3B–E**). Total Akt2 expression remained relatively constant across all groups. These *in vivo* findings demonstrate that GS-Rh2 suppresses Gab2 expression and Akt2 phosphorylation in intracranial glioma tissues, which is consistent with the *in vitro* cell experiment results.

### 3.6 GS-Rh2 inhibited subcutaneous glioma growth

**Figure 4:**
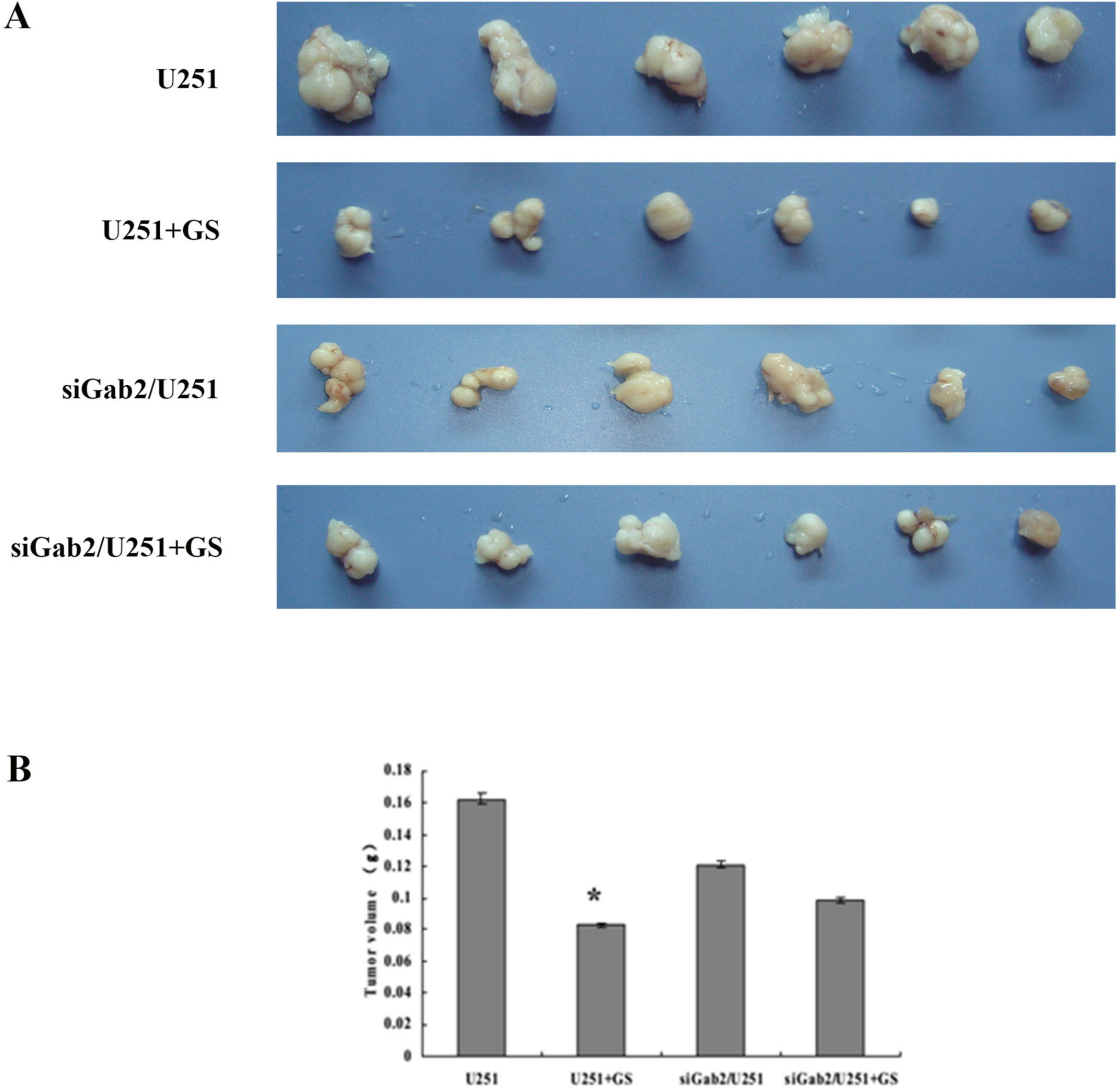
GS-Rh2 inhibits subcutaneous glioma growth in nude mice. **(A)** Representative photographs of excised tumor tissues from each group. **(B)** Quantification of tumor weight **(g)** in each group (n = 12, *P < 0.05 vs. U251 group, LSD test).

The subcutaneous xenograft results demonstrated that GS-Rh2 significantly inhibited the growth of U251-derived tumors in nude mice (n = 12, *P* < 0.05). The tumor weight in the siGab2/U251 group was significantly lower than that in the U251 group (*P* < 0.05); however, GS-Rh2 did not significantly further inhibit the growth of siGab2/U251-derived tumors (**Figures 4A, B**).

## 4 Discussion

The mechanism of action against glioma migration and invasion remains largely unexplored. While the anti-tumor effects of GS-Rh2 and the role of the Gab2/Akt signaling axis in glioma biology have been reported separately, the present study is the first to provide direct evidence that GS-Rh2 inhibits glioma cell migration and invasion specifically through the Gab2/Akt2 signaling pathway. This study thus bridges two previously independent lines of research, adding mechanistic insight into how a naturally derived compound targets a specific oncogenic signaling axis in glioblastoma.

Our results demonstrate that GS-Rh2 significantly inhibits U251 cell proliferation, migration, and invasion both *in vitro* and *in vivo*. This effect appears to be mediated through the downregulation of Gab2 expression and subsequent inhibition of Akt2 phosphorylation. *In vitro*, GS-Rh2 treatment resulted in a significant reduction in U251 cell proliferation, Gab2 expression, and Akt2 phosphorylation. Importantly, the inhibitory effects of GS-Rh2 on cell proliferation, migration, and invasion were substantially attenuated in Gab2-knockdown cells (siGab2/U251), suggesting that Gab2 plays a crucial role in mediating the anti-tumor effects of GS-Rh2. This aligns with our previous findings (29) demonstrating that Gab2 inhibition reduces U251 cell migration and invasion.

The lack of additional effect of GS-Rh2 in siGab2/U251 cells provides strong evidence that the primary mechanism of GS-Rh2 involves the Gab2/Akt2 pathway. Several studies have shown that Akt2 is associated with metastatic invasion of glioma (26, 27). Cui et al. (28) employed RNA interference to suppress Akt2 expression in malignant glioma cells and demonstrated that Akt2 silencing diminishes cell motility and invasion by reducing F-actin polymerization and impairing extracellular matrix adhesion. It is worth noting that total Akt2 protein levels remained relatively stable across experimental groups, indicating that GS-Rh2 specifically affects the phosphorylation status of Akt2 rather than its total expression, further supporting a post-translational regulatory mechanism via the Gab2 scaffold protein.

In the nude mouse intracranial xenograft model, GS-Rh2 significantly reduced the number of satellite tumor nodules in the brain, indicating substantial inhibition of tumor invasion. Western blot analysis of tumor tissues confirmed that GS-Rh2 reduced Gab2 expression and Akt2 phosphorylation *in vivo*. The GS-Rh2 dosage of 10 mg/kg used in this study was selected based on published pharmacological studies demonstrating effective anti-tumor activity at this dose range in murine models (7, 31), and is supported by pharmacokinetic data showing that GS-Rh2 crosses the blood-brain barrier and achieves biologically active concentrations in the central nervous system (4, 32). Similar to the *in vitro* results, the effect of GS-Rh2 was significantly reduced in the siGab2/U251 group, further supporting the critical role of Gab2 in mediating the anti-tumor activity of GS-Rh2.

Several limitations of this study should be acknowledged. First, the study used a single glioblastoma cell line (U251), and the *in vitro* findings require validation in additional glioblastoma cell lines and patient-derived xenograft models to enhance generalizability. The reliance on *in vitro* assays as the primary source of mechanistic evidence limits the translational conclusions that can be drawn. Second, although the *in vivo* studies showed promising results, tumor growth assessment was performed by direct measurement after excision, which may not fully capture the dynamics of intracranial tumor progression. Future studies should incorporate *in vivo* imaging modalities such as MRI or bioluminescence imaging for more accurate longitudinal evaluation of tumor burden. Third, larger sample sizes and longer follow-up periods are needed to fully assess the therapeutic potential and safety profile of GS-Rh2.

In conclusion, our findings suggest that GS-Rh2 exhibits potent anti-glioblastoma activity *in vitro* and *in vivo* by targeting the Gab2/Akt2 signaling pathway. These findings provide a rationale for further preclinical and clinical investigations of GS-Rh2 as a potential therapeutic agent for glioblastoma.

## 5 Conclusion

GS-Rh2 significantly inhibited Gab2 expression and Akt2 phosphorylation in U251 cells, resulting in decreased migration and invasion ability. In siGab2/U251 cells, where Gab2 expression was suppressed through RNA interference, the phosphorylation level of Akt2 was also significantly reduced, and GS-Rh2 did not exhibit significant additional inhibition of migration and invasion. Consequently, GS-Rh2 inhibits the proliferation, metastasis, and invasion of U251 glioma cells by suppressing Gab2 expression and Akt2 phosphorylation through the Gab2/Akt2 signaling pathway.

## Data Availability

The raw data supporting the conclusions of this article will be made available by the authors, without undue reservation.
